# The effect of high-volume image-guided injection in the chronic non-insertional Achilles tendinopathy: a retrospective case series

**DOI:** 10.1186/s40634-020-00264-4

**Published:** 2020-06-27

**Authors:** Torsten Grønbech Nielsen, Lene Lindberg Miller, Bjarne Mygind-Klavsen, Martin Lind

**Affiliations:** Orthopedic Department, Aarhus University Hospital, Palle Juul-Jensens Boulevard 99, 8200 Aarhus N, Denmark

**Keywords:** High-volume image-guided injection, HVIGI, Achilles, Tendinopathy, Achilles tendon, Eccentric training

## Abstract

**Purpose:**

To evaluate if High-volume Image-guided Injection (HVIGI)-treatment for chronic mid-portion Achilles tendinopathy (AT) improve function and reduce pain at 12-months follow-up.

**Methods:**

Patients with resistant mid-portion AT who failed to improve after a three-month eccentric loading program were included in the study. Maximal tendon thickness was assessed with ultrasound. All patients were injected with 10 mL of 0.5% Marcaine, 0.5 mL Triamcinolonacetonid (40 mg/mL) and 40 mL of 0.9% NaCl saline solution under real-time ultrasound-guidance and high pressure. All outcome measures were recorded at baseline and 12 months. A standardized eccentric rehabilitation protocol was prescribed after HVIGI-treatment. Clinical outcome was assessed with the Victorian Institute of Sports Assessment-Achilles tendon questionnaire (VISA-A) and statistically analyses were performed.

**Results:**

The study included 30 single treatment HVIGI procedures in AT in 28 patients (23 men, 5 women) with a mean age of 45.1 (range 16–63). The mean duration of symptoms before HVIGI was 37 months. The baseline VISA-A score of 50 ± 15 (range 14–74) improved to 61 ± 21 (range 31–94) after 1 year (*p* = 0.04). Of the 30 AT procedures 10 patients (11 AT) were not satisfied after the initial HVIGI procedure. Of these, 8 patients (9 AT) needed additional HVIGI and two patients needed surgery. Of the remaining 18 patients (19 AT), 10 patients had more than a 10-point improvement in the VISA-A score after 1 year.

**Conclusions:**

In this retrospective case-study, only 10 patients (33%) did benefit of a single HVIGI treatment at 12-months and an 11-point significant improvement was seen at on the VISA-A score.

## Background

Achilles tendinopathy (AT) is a common overuse injury among runners. Elite long-distance runners have a 52% risk of developing AT in their life span [1]. The prevalence and incident rate in the general population is 5.2 and 1.7–2.3 per 1000 registered patients respectively [2, 3]. AT is a degenerative condition and is characterized by a neovascularisation of the Achilles tendon. The area of degeneration occurs proximal of the tendon insertion into the calcaneus in the mid-portion of the tendon [4]. The neurovascular ingrowth is mainly located at the ventral part of the Achilles tendon [4]. The neovascularisation and accompanying nerve -ingrowth is found in patients with painful AT, but not in normal tendons [4, 5].

Various treatment strategies for AT exist in the literature. A meta-analysis from 2012 advocated for eccentric training [6]. But other treatment modalities including shock wave, dry needling, corticoid injection, load modification etc. have been performed with various results over the past decades [7–10]. No golden standard regime exist for patients with AT.

In 2008, Chan et al. performed the first High-volume Image-guided Injection (HVIGI) study for chronic non-insertional AT. HVIGI was used in a cohort of patients who failed a three-month guided eccentric training program (ET). The mechanism behind the effect of HVIGI-treatment is believed to be mechanical stretching, breaking or occluding the neo-vessels and the accompanying nerve ingrowth [11]. This is believed to reduce the pain of the tendinopathy.

The aim of the study was to evaluate if HVIGI-treatment for chronic mid-portion AT improve function and reduce pain at 12-months follow-up.

It was hypothesised that HVIGI would result in clinically relevant improvements of symptoms and function for chronic non-insertional Achilles tendinopathy after one-year and that most of the patients would benefit from HVIGI treatment.

## Methods

In this retrospective case series of 42 single HVIGI procedures followed prospectively were performed between September 2013 and November 2016. Patients were included if they had a diagnosis of tendinopathy (here defined by pain in the Achilles tendon proximal to the insertion site on calcaneus, thickness of the tendon [> 6 mm] and neovascularisation in the tendon measured on sonography) in the mid-portion of the Achilles tendon and had failed a three-month ET program. Patients were excluded if they have had prior HVIGI treatment or were lost to follow up.

The Local Ethics Committee was contacted and determined that an approval for this study was not required (1–10–72-1-19). All data was managed with strict confidentiality and data has been anonymised prior to analysis. Three skilled orthopaedic surgeons diagnosed the patients on the basis of their medical history, physical examination and ultrasound evaluation.

### Intervention

#### Injection

Patients were placed in the prone position. To avoid the nervus suralis, the needle was inserted between Kager’s fat pad and the anterior aspect of the AT using an aseptic technique. The injection was thus extra tendinous. AT injection was performed with 10 mL of 0.5% Marcaine, 0.5 mL Triamcinolonacetonid (40 mg/mL) and 40 mL of 0.9% NaCl saline solution under real-time ultrasound guidance aiming at the area of maximal neovascularisation.

#### Rehabilitation

In the first 72 h after the injection, patients were only allowed to participate in the normal activities of daily living. Running, jumping or heavy resistance training were prohibited. After 72 h, patients were allowed to begin ET as described by Alfredson et al. [12]. A two-page leaflet with exercises and guidelines was given to the patient. Patients knew the rehabilitation due a previously failed three-month rehabilitation program. If patients had the intension of returning to running and were pain-free, they were educated in a graded running program starting 1 month after HVIGI treatment.

### Clinical outcome measure

VISA-A [13, 14] was used as a primary outcome measure and was recorded at baseline and 12 months. This score ranged from 0 to 100, where 0 was worst and 100 was asymptomatic. Primary outcome was the delta value between baseline and the 12-month follow-up.

A successful outcome was defined as one single HVIGI treatment, no subsequent conversion to surgery and a 10-points improvement at the VISA-A at the 12-month follow-up.

### Radiological evaluation

Maximal tendon thickness and neovascularisation were assessed with ultrasound and power Doppler in the prone position with the patient’s feet hanging at the end of the table. The Achilles tendon was scanned in both transverse and longitudinal planes. Radiological evaluation was performed at baseline and 12-month follow-up.

### Statistical analysis

Descriptive statistics were calculated. Continuous data were presented as mean ± standard deviation (SD). Categorical data were presented as quantity and proportions (%). Normality was tested by qq-plots. All data were normally distributed, and the student’s T-test was used to compare differences between time-points. Minimal clinical important difference (MCID) was set at 10 points. P-values below 0.05 were considered significant [15]. All data was analysed in MS Excel 2010 version 14.0.7237.5000 (32-bit) and STATA 16.0 software (StataCorp, College Station, TX, USA).

## Results

**Fig. 1:**
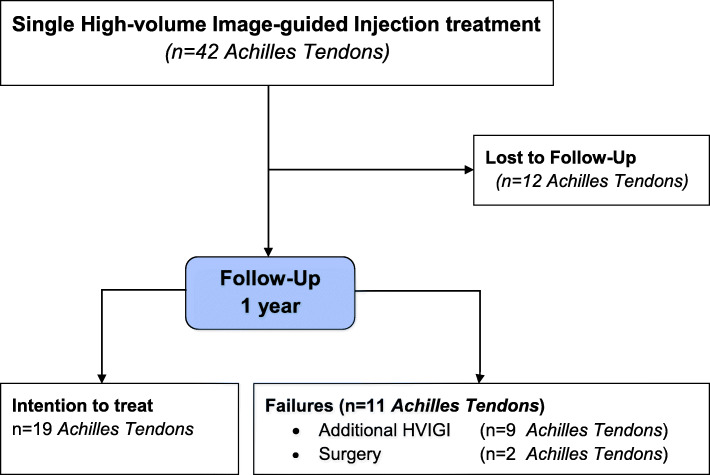
Flow Chart

Forty-two single treatment HVIGI procedures were performed in the period 2013–2016. The study included 30 AT in 28 patients (23 men, 5 women) (Fig. 1). The mean age was 45.1 years (range 16–63). The mean duration of symptoms before HVIGI was 37 months (range 3–180).

Patient characteristics are listed in Table 1. Some patients had tried several other conservative treatments apart from ET before HVIGI including corticosteroid injection, extracorporal shock-wave therapy or acupuncture (Table 1).

**Table 1:** Patients demography

HVIGI procedures/patients, n	30/28
Men/Women, %	82/18
Mean age in years (range)	45.1 (16–63)
Mean pain duration in months (range)	37 (3–180)
Intervention before HVIGI	
• Corticosteroid, n (%)	18 (60%)
• Shock Wave therapy, n (%)	5 (17%)
• Acupuncture, n (%)	1 (3%)

The baseline VISA-A score of 50 ± 15 (range 14–74) improved significantly to 61 ± 21 (range 31–94) at the one-year follow-up (*p* = 0.04). VISA-A scores are listed in Table 2. Ten patients (33%) having a single HVIGI treatment had a 10-point improvement.

The thickness of the Achilles tendon measured at baseline was 8.8 ± 2.0 mm compared to 8.9 ± 3.7 mm at the one-year follow-up (*p* = 0.94) (Table 2).

**Table 2:** VISA-A and Achilles tendon thickness

	Baseline	1 Year	***p-value***
Achilles Tendons/Patients	30 / 28	19 / 18	
VISA-A completeness, n (%)	30 (100)	19 (100)	
VISA-A, mean	50 ± 15	61 ± 21^a^	*0.04*
> 10 points improvement, n (%)		10 (33)	
Achilles tendon thickness, mm	8.8 ± 2.0	8.9 ± 3.7	*0.94*

Results are presented as mean-values with standard deviation (±SD). ^a^Significant improvement from baseline

Of the 42 AT treated for a single treatment with HVIGI 11 patients (12 AT (29%)) were lost to follow up.

No significant differences at baseline in age, mean duration, VISA-A or tendon thickness were found when comparing the group of patients lost to follow up and the patients included in this study.

Of the remaining 30 AT (28 patients), 10 patients (11 AT (37%)) were not satisfied after the single HVIGI procedure and required further treatment. Of these, 8 patients (9 AT (30%)) had additional HVIGI and two patients had surgery (7%).

No complications in relation to HVIGI treatment were observed.

## Discussion

The primary finding of the present study was that a successful outcome after a single HVIGI treatment in patients with Achilles tendinopathy who had previously failed previous training treatment was seen in 33% of patients after 1 year. A significant improvement was demonstrated compared to the baseline VISA-A score. The improvement in the VISA-A score from baseline to the 12-month follow-up was 11 points. This improvement is different from previous studies presenting one-year results after HVIGI (Table 3). Maffulli, Wheeler and Abdulhussein found an improvement from baseline to 12 months in their studies at 33, 41 and 40 points, respectively [18, 19, 21]. A difference between these studies and the present study could be the long duration of symptoms in our patient cohort. Thirty-five months was the time between the onset of Achilles symptoms and HVIGI procedure compared to 13–57 weeks (3–13 months) in the above-mentioned studies. However, in these studies, all patients had also failed a three-month ET and other interventions, which is comparable to the present study.

**Table 3:** Studies presenting data on High-volume Image-guided Injection (HVIGI)

	Pts (n)	Age (y)	DoS (wk)	Post (wk)	US Δ (mm)	VISA-A pre	VISA-A post	VISA-A Δ	ET failed
**Chan** [11] **(2008)**	30	37	36	30	/	44 ± 18	76 ± 25	32	YES
**Humphrey** [16] **(2010)**	11	44	220	3	1.1	46 ± 15	84 ± 11	38	YES
**Resteghini** [17] **(2012)**	32	40	73	12	2.7	37	66	29	YES
**Maffulli** [18] **(2013)**	94	38	57	52	1.8	42 ± 23	75 ± 21	33	YES
**Wheeler** [19] **(2014)**	16	53	31	52	/	31 ± 22	72 ± 26	41	YES
**Wheeler** [20] **(2016)**	18	53	/	40	/	30 ± 21	64 ± 28	33	/
**Abdulhussein** [21] **(2017)**	12	46	13	52	/	43 ± 9	83 ± 12	40	YES
**Boesen** [9] **(2017)**	19	42	25	24	1.1	53	75	22	NO
**Boesen** [22] **(2019)**	14	43	44	24	1.2	52 ± 14	78 ± 5	26	NO

VISA-A are presented as mean-values with standard deviation (±SD). *Pts* patients, *DoS* Duration of symptoms, *Post* Latest follow-up, *US* tendon thickness between baseline and Latest follow-up, Δ = improvement from pre to post, ET failed = ET failed before HVIGI, /=unknown

All studies in Table 3 demonstrate significant improvement at both short- and longer-term follow-up on the VISA-A score. Several differences between these studies have been observed: variation in age group (37–53 years), duration of symptoms before inclusion (13–220 weeks) and final follow-up time (3–52 weeks). Furthermore, differences in inclusion criteria are observed; where Boesen et al. [9, 22] used HVIGI as the initial treatment, other studies only included patients who had failed a three-month eccentric training regimen. No difference between injection techniques and rehabilitation regimens was observed in the studies listed in Table 3.

Maffulli et al. demonstrated in their cohort that patients who had corticoid injections as part of the previous treatment before HVIGI had lower outcome improvement than the rest of the cohort (61.8 on the VISA-A) [18]. In the present study, 60% patients had prior to the HVIGI treatment either corticosteroid injection (*n* = 18), extracorporal shock wave (*n* = 5) acupuncture (*n* = 1) or more than one intervention. Combining this issue of a high proportion of our cohort having previous corticosteroid treatment with the long duration of symptoms of mean 37 months, indicates that the HVIGI treatment in the present study served therefore as a salvage procedure for AT when other treatments had failed. This could explain the low treatment response rate compared to previous studies in patients with less chronic conditions.

In the present study, a successful outcome was defined as one single HVIGI treatment, no subsequent conversion to surgery and a 10 point improvement at the VISA-A at the 12-month follow-up. Some studies have used patient satisfaction questionnaires to define a successful outcome. Boesen et al. used a dichotomous ‘participant satisfaction questionnaire’ in their studies and respectively demonstrated a 63% and 75% rate of overall treatment satisfaction among the patients after 24 weeks [9, 22]. In another study, the rate of return to sports was measured after 12 months; 61% of patients returned to same level or above as before injury [21]. An other study has mentioned a VISA-A score above 96 points as normal values for healthy patients [14]. None of the patients in the present study were demonstrated such a healthy VISA-A score after 12 months. No studies have calculated MCID for VISA-A in patients with mid-portion AT. MCID scores of 10 and 12 points in the VISA-A score have been used in previous studies [15, 23].

Maffulli et al. reported that 45 patients (almost 50%) needed an additional HVIGI treatment within the first 6 weeks after first HVIGI treatment. Eight of those required surgery at a later point. Despite that, they demonstrated a return to a desired level of sports activity in 68% of the patients [18].

Other injective modalities have been used in treatment of AT. Boesen et al. found no difference in VISA-A score at 24 weeks follow-up in a randomised double-blinded study comparing HVIGI and platelet-rich plasma [9]. Corticosteroid injections are commonly used for AT and demonstrate short-time efficacy, but symptom recurrence in the long term [23, 24].

In the present study, no impact on Achilles tendon thickness after HVIGI treatment after 12 months was found. Other HVIGI studies have found reduced thickness of 1.1 to 2.7 mm at various points [9, 16–18, 22]. A 1.8 mm reduction in tendon thickness was found in the only study presenting Achilles tendon thickness at 12 months [18].

It appears, based on the present study, that patients with long-lasting AT who have failed either a three-month ET, corticosteroid injection or extracorporal shock-wave treatment will only have a 33% chance of a clinically relevant improvement in subjective outcome after a single HVIGI treatment after 1 year. These results indicate that patients with chronic AT and failed previous treatment should be counselled about the limited success rate of further treatment with HVIGI. The HVIGI treatment is found safe, due to no complications observed regarding this cohort.

Several limitations were acknowledged in this study. The most important were related to the absence of a control group and the lack of a training dairy. Of the patients did perform the exercises as prescribed was unknown. Vascularisation and neovascularisation were not measured at baseline and 12 months. Functional testing such as; gait analysis, strength assessment etc. could be beneficial in the analysis of the outcome after the HVIGI intervention.

## Conclusion

In this retrospective case-study, 33% of the patients with chronic mid-portion AT who had failed guided ET did benefit of a single HVIGI treatment at one-year follow-up. An 11-point significant improvement was seen at 12-month follow-up on the VISA-A score.

## Data Availability

The datasets used and/or analysed during the current study are available from the corresponding author on reasonable request.

## Abbreviations

HVIGI: High-volume Image-guided Injection
AT: Achilles tendinopathy
VISA-A: Victorian Institute of Sports Assessment-Achilles tendon questionnaire
ET: Eccentric training
MCID: Minimal clinical important difference
